# Two Cases of Eyelid Leukoderma Associated With Eyelash Extension

**DOI:** 10.1111/jocd.16564

**Published:** 2024-09-17

**Authors:** Jing Zhu, Lingling Luo, Youming Guo, Chengrang Li

**Affiliations:** ^1^ Hospital for Skin Disease Institute of Dermatology, Chinese Academy of Medical Sciences & Peking Union Medical College Nanjing China

Dear Editor,

1

We describe two cases of eyelid leukoderma in two young women, both of whom had a history of eyelash extensions before the appearance of depigmentation. We assumed the eyelid leukoderma of the two patients was associated with eyelash extension glue. There is no existing literature related to chemical leukoderma induced by eyelash extension. We reported the two cases here, and dermatologists should pay more attention to this type of chemical leukoderma.

## 2 Case Report

**FIGURE 1:**
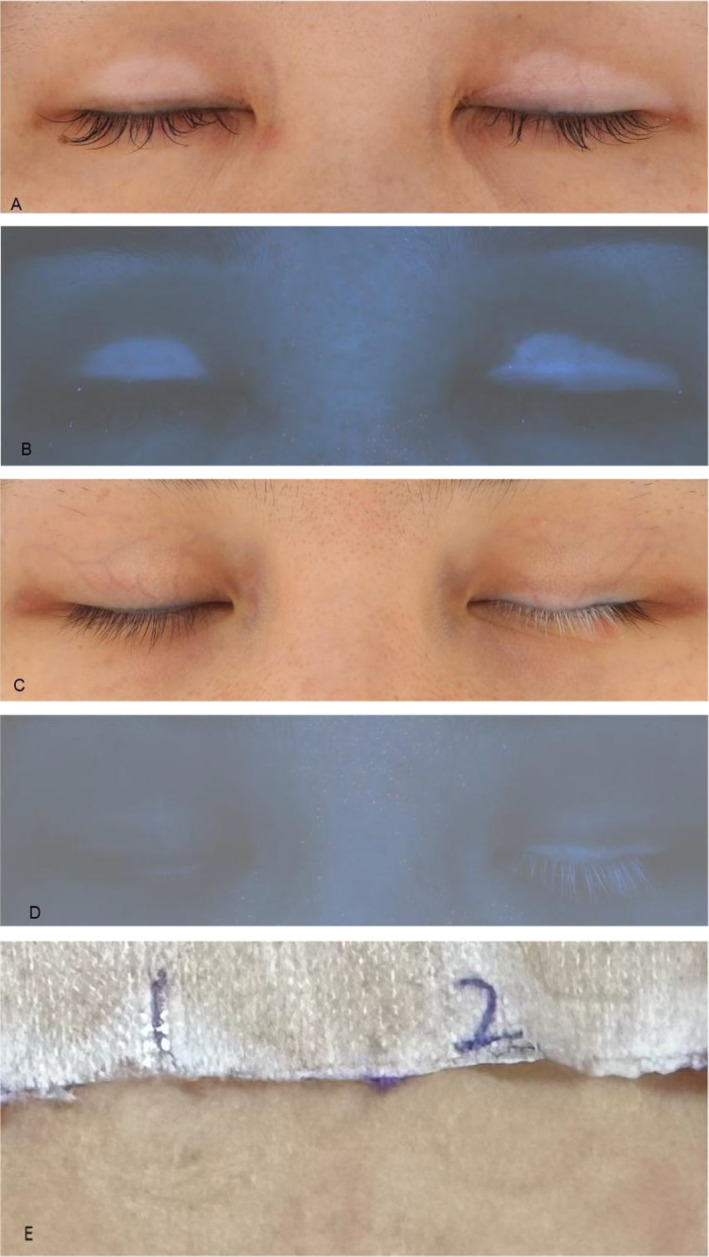
(A) The depigmentation patches were located on the skin of both eyelids. (B) Under Wood's lamp, the depigmentation patches were grayish‐white with clear boundaries. (C) The depigmentation patches mainly distributed on the skin of the left eyelid and eyelash. (D) Wood's lamp examination showed the involvement of the eyelashes on both sides. (E) The patch test for the glue of Patient 2 is negative.

Patient 1 was a 38‐year‐old woman who visited our clinic and complained of bilateral eyelid depigmentation patches for 1 month. She had no discomfort, such as itching or erythema appeared before the depigmentation. Our physical examination revealed depigmentation patches on both eyelids (Figure 1A) and no other depigmented areas. Under Wood's lamp, the patches were grayish‐white with clear boundaries (Figure 1B). We conducted comprehensive laboratory testing which showed higher‐than‐average thyroglobulin and thyroid peroxidase antibody titers. Antinuclear antibody tests were negative. The patient had performed the eyelash extension using chemical glue 1 month before the eyelid depigmentation patches appeared. It was her first time using glue to make the eyelash extension. Based on these findings, we diagnosed eyelid leukoderma and believed it might be associated with eyelash extension. We initiated a treatment plan involving the topical application of 0.03% tacrolimus cream, which reduced the depigmentation patch size.

Patient 2 was also a young woman who presented with bilateral eyelid depigmentation that had been present for 2 years. She also did not report itching or erythema. The depigmentation on the skin of the left eyelid was identifiable (Figure 1C), while Wood's lamp examination accentuated the depigmented eyelashes of the other side (Figure 1D). This patient also had undergone eyelash extensions with chemical glue several times with a beautician's help, and no depigmentation happened. The depigmentation appeared 2 weeks later when she started making eyelash extensions with a new glue herself without the beautician's help. Then, she stopped any eyelash extensions and went to the hospital for therapy. Patch testing was applied on the patient's upper back with the original glue sample she had used for eyelash extension. The patch test result for the glue she had used was negative (Figure 1E). We also diagnosed eyelid leukoderma.

## 3 Discussion

Many factors, such as autoimmune‐mediated melanocyte destruction, skin senescence, and chemicals, can cause acquired skin depigmentation [1]. For instance, vitiligo is caused by the autoimmune targeting of melanocytes and is characterized by well‐demarcated patches of different sizes and shapes [2]. Chemical leukoderma can be induced by natural extracts found in cosmetics, such as monobenzyl hydroquinone [3] and rhododendrol [4], resulting in the appearance of leukoderma at the application site.

Both patients' eyelid depigmentation happened with a history of eyelash extensions, which need chemical glue. It was easy to make us think of the depigmentation related to the chemical glue. We found that eyelash extension glue always contains acrylates component. Acrylates, which frequently contain allergens, can sensitize individuals through nail products and medical devices. Several reports marked that beauticians and hairdressers developing occupational allergic contact dermatitis induced by acrylates, which puts them at risk and their clients can develop facial dermatitis or eyelid dermatitis [5]. There are several case reports even address acquired leukoderma following patch testing with an acrylate series [6, 7]. Both patients did not experience itching or erythema before depigmentation onset, making it unlikely could that the leukoderma be the result of post‐inflammatory hypopigmentation. Chemical leukoderma denotes acquired hypopigmentation due to repeated exposure to specific chemical compounds and often mimics idiopathic vitiligo. Though the first patient had done eyelash extension only once, the glue had been present for a period before the depigmentation appeared. In chemical leukoderma, the depigmentation is limited to the site of chemical contact. However, initial contact sites can gradually expand to the whole body and become typical non‐segmental vitiligo, indicating that some chemical antigens can act as environmental triggers or haptens to induce typical vitiligo3. Certain chemicals are cytotoxic to melanocytes due to competitive inhibition of tyrosinase [8]. Moreover, studies have suggested that dendritic cells play a role in the spread of leukoderma [9]. However, only one of the patients had done patch testing, and the result was negative. The glue used to perform the eyelash extension was present on the eyelash for a period. The negative outcome of the patch test means the glue did not trigger contact dermatitis. It cannot rule out the effect of persistent presence on eyelashes or slow repeated contact with the skin, such as depigmentation.

In conclusion, we believe both patients' leukoderma was associated with eyelash extensions. Whether chemicals such as acrylic compounds can promote the apoptosis of melanocytes and whether this is related to intrinsic defects requires further investigation.

## Conflicts of Interest

The authors declare no conflicts of interest.

## Data Availability

The data that support the findings of this study are available from the corresponding author upon reasonable request.
